# Editorial for the Special Issue on “Fundamentals of Adsorbents–Synthesis, Characterization, Properties, and Application”

**DOI:** 10.3390/ma15062122

**Published:** 2022-03-14

**Authors:** Durga Parajuli

**Affiliations:** Nanomaterials Research Institute, National Institute of Advanced Industrial Science and Technology (AIST), 1-1-1 Higashi, Tsukuba 305-8565, Japan; parajuli.durga@aist.go.jp

This Special Issue covers the widely studied topic of “Adsorbents”, materials that are known to uptake ions and molecules from water or air. With nine original articles kindly contributed from a wide spectrum, this Issue compiles some of the new and ongoing trends in this area of research. Two of the articles are concerned with the characterization and application of inorganic porous materials [1,2]. Three papers discuss synthetic polymers: the synthesis or recycling of the polymers and their positive and negative impact in the environment [3,4,5]. The term carbon itself represents a centuries-old adsorbent. Three articles cover the synthesis, characterization, and application of nano or modified carbons [6,7,8]. Similarly, the study on the application of a municipal solid waste compost as an adsorbent is very interesting [9].

The adsorption method has long been used in both air and solution systems. For issues such as rare element recovery/recycling, resolving the freshwater scarcity issue, heavy metal and radioisotope decontamination, biomass adsorbents, porous coordination polymers, organic resins, inorganic complexes, and so on have been extensively studied. In addition, from freshwater and seawater systems to organic–aqueous mixtures, the need for an adequate adsorbent is immense. Articles in this Issue cover topics such as the decontamination of organic dyes, pesticides, or heavy metals from solution or soil; the recovery of valuable elements from seawater; the removal of CO_2_; and the assessment of the detrimental effects of the microplastics.

Synthetic polymers and biomasses are the main substrates or feed materials for obtaining a target specific modified adsorbent. Natural zeolites are good alternatives, although this group of materials is not known to show specific selectivity for given any ions or molecules. Several kinds of mixed metal oxides exist in nature. Kokkinos et al. studied heavy toxic metals’ (Pb, Cd) removal potential from tetravalent manganese feroxyhyte, TMFx [1]. The successful engagement of the divalent Ca ion to its surface has greatly enhanced the efficiency of this material. Target specific modification, such as surface charge, exchangeable ions, size, porosity, etc., has multiplied the dimensions of the research on adsorption/adsorbents. A highly selective adsorption of K from seawater in the presence of approximately 46 times relative Na concentrations reflects the importance of a target specific adsorption material [2]. In this case, the material releases equimolar Na into the seawater while adsorbing K. Although the addition of a little Na into the saline water does not make any impact, separating K either as a resource or for the purpose of obtaining high purity Na compounds from seawater makes a big difference. 

The use of synthetic polymers as adsorbents is diverse. The concept of recycling polyvinylchloride or high-density polystyrene as adsorbents, as introduced by Hermosillo-Nevarez et al., has added another potential application of these widely used materials [3]. Similar to their use for malathion decontamination, possibilities for their further application might be expanded with slight modification, for example, through the enhancement the porosity or the introduction of ion-selective functional groups, as described by Paczkowski et al. [4]. In the context of synthetic polymers, the issue of microplastics cannot be understated. Perhaps the use of recycled plastics and mindful end-treatment are the keys to controlling the risks [5]. Additionally, promoting the use of plant-based adsorbents would be a major sustainable move in real sense.

As the plant kingdom is diverse, feed materials for obtaining the plant-based adsorbents can vary widely. Studies on the preparation and characterization of carbon adsorbents from available plant resources have been underway for a long time. Three similar works are included in this issue: obtaining nanoporous carbon from male oil palm flowers [6], biochar from bamboo [7], and activated carbon from rubber-seed shells [8]. These materials were studied either in aqueous systems or through gas adsorption. This means that by varying the method of preparation/modification plant-based carbon adsorbents can be obtained. An interesting addition to the use of plants as adsorbents is the utilization of compost [9]. There is merit to a discussion on the leaching from compost that occurs during its use in water containing systems; however, the removal of toxins from soil, for example, should not be considered too worrisome. 

Although the issue could not progress as expected because of the global COVID-19 pandemic, which greatly affected lab activities, the presented articles compile some interesting developments in the field of the development and characterization of adsorbents. I would like to express my sincere thanks to the contributors and reviewers for assisting with their expertise and time. I am also thankful to the Materials editorial team for their continuous support.
